# The Vickers SP 120 analyser: an instrument evaluation

**DOI:** 10.1155/S1463924678000103

**Published:** 1978

**Authors:** B. P. Ager, R. Gentle, E. W. Ingarfill, A. L. Tárnoky

**Affiliations:** 1 The Department of Health and Social Security London UK; 2 The Department of Clinical Chemistry Royal Berkshire Hospital Reading UK; 3 Scientific and Technical Branch Department of Health and Social Security 14 Russell Square London WCIB 5EP UK


### The Vickers SP 120 analyser: an instrument evaluation

**B. P. Ager** †
*The Department of Health and Social Security, London, UK*

**R. Gentle, E. W. Ingarfill and A. L. Tárnoky**
*The Department of Clinical Chemistry, Royal Berkshire Hospital, Reading, UK.*

**Introduction**
For logistic, if not economic, reasons the larger clinical chemistry departments handle much of their work by automated multi-channel analysis. Their commonest purpose-built equipment is the Technicon* SMA 12/60 analyser, first introduced into the United Kingdom in 1969. It can process up to 60 samples an hour and provides twelve analytical results on each.

This speed of working is now becoming too slow: in some hospitals an increasing workload is beginning to exceed the capacity of the SMA 12/60 or its recent variants and, even where it does not, staff spending less time with faster equipment could then be redeployed. The Vickers** SP 120 analyser is designed to meet present-day needs. It is an attachment to the SMA 12/60, doubles its output and in so doing reduces its reagent consumption and sample size. We report on its performance.

This instrument will always be used in conjunction with the Technicon SMA series for which it was designed. Its attachment nature therefore simplified our task in several respects to a straight-forward comparison of the 12/60 + 120 complex with the original 12/60, already well known — Broughton et al [1]. In other respects our evaluation was based on an accepted schedule recommended by Broughton et al [2] with help from a similar report by Schwartz et al [3].

### Materials and methods

### The Technicon SMA 12/60

This instrument has been the subject of numerous reports Broughton et al [1], Technicon Bibliography [4] and needs no further description. Standard Technicon analytical methodologies are employed at the Royal Berkshire Hospital with the exception of albumin (bromocresol green) Northam et al [5], alkaline phosphatase (disodium phenyl phosphate) Robinson et al [6], phosphate (vanadate) Goldberg et al [7], and the use of non-flowrated pump tubes.

### The Vickers SP 120

This is a computer-controlled system designed to monitor and control the Technicon instrument which is attached to it. The system at the Royal Berkshire Hospital consists of an SP 120 process controller (made up of a Digital Equipment Corporation PDP 8A computer, XEBEC flexible disc system, Hewlett Packard scope and Vickers Medical analog electronics) a Centronics 308 teleprinter capable of printing 165 characters per second, 80 columns per line: a Pitney-Bowes laser beam bar code reader for sample identification: and a Beehive Super-Bee visual display unit. The process controller takes over the functions of the 12/60 programmer and function monitor, while the analog presentation of results on chart paper is replaced by a printout from the Centronics printer. The system is fitted to an existing SMA by plugging into each of the colorimeters and the sampler, the programmer having been disconnected. The 12/60 programmer, chart recorder and function monitor are switched off. The SP 120 eliminates manual phasing and calculates a sample "inter-reaction" correction to compensate for carryover.

**Operation**
Each day the process controller calculates the inter-reaction corrections and the phasing times for each channel, from an "Initial Tray" loaded with phasing solution and high and low sera. The corrections and times taken from this tray are used throughout the day or until another Initial Tray is run.

The analysis trays are run with two identical calibration standards at the beginning and the end of each tray and the results are calculated on the mean of these standards. When analysis of the first tray is complete, the teleprinter puts out a provisional report of the results and controls. Error messages are printed out at any stage during the run to indicate poor peaks or results beyond the scale of the channel and results outside the laboratory limits are flagged. Poor phasing during the Initial Tray run is also flagged: the tray is then run again, or phase times are edited manually.

The day's run is started by the requisite "CHEM" program processing an Initial Tray through the instrument. It takes the timing for all subsequent readings from its first signal, the phasing solution in cup 1. (Phasing solution: Wellcomtrol "Autoset H Unassayed" — Wellcome Reagents Ltd, for 25 ml of solution, dissolved instead of 18 ml of the bicarbonate diluent + 2 ml of the 1000 $\mu$g% iron stock standard solution of the routine analytical method + 20 ul Aspartate Transaminase Suspension — Sigma cat No G-2751 + 25 ul Technicon red chart recorder ink + 25 $\mu$l Sheaffer Skrip or similar blue ink).

The other cups contain the prescribed sequence of high and low sera or plasmas from which the program calculates percentage interactions. (High and low serum or plasma: a mixture of bovine with some human material, and an aqueous 1:10 dilution of this. The composition of this pooled serum or plasma varies from day to day, and its actual concentrations are immaterial. Typical values would be: Total Protein 85 g/l, Albumin 43 g/l, Calcium 2.9 mmol/l, Phosphate 2.2 mmol/l, Cholesterol 5.2 mmol/l, Iron 36 $\mu$mol/l, Urate 0.53 mmol/l, Creatinine 700 $\mu$mol/l, Total Bilirubin 100 $\mu$mol/l, Alkaline Phosphatase 200 IU/l, Lactate Dehydrogenase 550 IU/l, Aspartate Transaminase 100 IU/l).

Trays are meanwhile loaded with test samples, each cup carrying a barcoded identity label. These trays are then run. They include calibration standards and, in cup positions 19 and 38, control sera (Ortho Diagnostics unassayed normal control serum — Ortho Diagnostics Inc).

The standard and test voltages from the colorimeters are stored until the tray is finished. The program then calculates voltages for the standard and concentrations for the test and control samples, identified by the tray number, cup number and

†Address for correspondence: Scientific and Technical Branch, Department of Health and Social Security, 14 Russell Square, London WC1B 5EP, UK.
*Technicon Instruments Co Ltd, Evans House, Hamilton Close, Basingstoke, Hants RG21 2YE
**Vickers Ltd, Medical Engineering, Priestley Road, Basingstoke, Hants RG24 9NP

the sample number read by the laser beam. These results are the material of the provisional reports, printed individually per patient who is identified only by these three numbers.

With a remote terminal the patients' identification data can be typed in and, on a subsequent command, merged with the analytical data and again printed out, this time as fully identified patient reports.

The quality control program is run at the end of the day's analyses, to give mean, SD, CV and SE of the control sera tested that day. The same calculations can also be carried out for the patient data and this operation may be restricted by using exclusion limits.

A one-day summary of results is also printed, in alphabetical order of patients.

A 30-minute wash-through period ends the working day.

### Clinical laboratory evaluation

The basic difference between the two systems is a shorter sampling time in the SP 120 complex, less than half that of the SMA 12/60, with consequent changes in the final colorimetric peak and the method required for reading it. These therefore were the aspects examined.

The sampling and wash times for both systems were measured with a stopwatch and compared with the theoretical values taken from the programming. *Sample volumes* — a direct function of sampling times — were calculated for each system from the weighed contents of 40 cups. The proportional volume of reagents used was calculated for two typical runs of 200 patients' samples.

*Linearity* was measured in a series of samples placed in ascending and descending order of concentrations. Adjoining duplicates of these samples were loaded and analysed once each on 3 days as part of runs standardised in the usual way, and the second of each duplicate pair of results was taken. For most channels the serum was Wellcomtrol Autoset H, dissolved in less than the prescribed volume, and its aqueous dilutions to 80, 50, 40, 20 and 10 per cent. For the enzyme channels, sera from patients were mixed in known proportions to give a series of values.

Precision measurements, both intra-batch and inter-batch were made by a 20-day comparison of 10 sets of results from each system at high, medium and low levels using sera available from Technicon Co Ltd., for SMA post-maintenance performance checks. Drift was not measured separately but deduced from the precision measurements.

A high-concentration and a low-concentration serum were used to observe *carryover*. Wellcomtrol Autoset H was taken for the high serum H and an aqueous 1:1 dilution of Ortho Diagnostic normal control serum as the low serum L. This measurement was taken on four days, on days 1 and 3 of which one sample plate of four low — high sequences (samples loaded as LLLLHHHHLLLLHHHHLLLLHHHHLLLLHHHH) were analysed, while on the 2nd and 4th day the sequences were reversed. There was one such run on both systems each day.

### Results

The *sampling time* of the SMA 12/60 is programmed as 54 s, its *wash time* as 6 s, and the SP 120 program cuts these times to 25 s for sampling and a 5 s wash. The values found in practice are slightly but measurably different, 53 s and 4 s for the SMA 12/60 cut to 24 s and 3 s by the SP 120. *The sample volumes* aspirated during these times (mean ±SD of 40 measurements each) are correspondingly reduced from 2.05 ± 0.012 $cm^3$ to 0.94 ± 0.022 $cm^3$, a saving of over half the sample, with a more variable uptake (CV 0.59 for the SMA 12/60, 2.30 for the SP 120) which, given steady state conditions, does not seem to affect the overall precision.

There is also a saving in reagents used with these samples, though this is partly offset by the longer setting-up time required by the SP 120 system (about 40 min, twice that of the SMA 12/60 alone). A typical troublefree run of setting up the combined system and processing a batch of 200 samples with, in addition, their complement of standard and control sera, will require 66.5% of the reagent volume consumed for the same load by the SMA 12/60.

**Table 1 Linearity of SP 120 - SMA 12/60 complex analyser**

| Analysis | Units | R | Intercept | Slope | SD of Points |
|---|---|---|---|---|---|
| Total protein | g/l | 1.000 | 0.2 | 1.00 | 1.24 |
| Albumin | g/l | 0.988 | 7.2 | 0.95 | 5.83 |
| Calcium | mmol/l | 0.999 | 0 | 1.00 | 1.21 |
| Phosphate | mmol/l | 0.999 | 0 | 0.99 | 1.75 |
| Cholesterol | mmol/l | 0.993 | 0.7 | 0.97 | 4.52 |
| Iron | μmol/l | 0.990 | -7.2 | 1.07 | 5.78 |
| Urate | mmol/l | 0.999 | 0 | 0.99 | 1.51 |
| Creatinine | μmol/l | 0.999 | 38.3 | 0.97 | 1.92 |
| Total bilirubin | μmol/l | 1.000 | -2.8 | 1.01 | 1.21 |
| Alkaline phosphatase | IU/l | 1.000 | -4.9 | 1.01 | 9.23 |
| LDH | IU/l | 0.999 | 14.9 | 0.99 | 2.07 |
| AST | IU/l | 0.994 | 3.0 | 1.05 | 4.74 |

**Table 2 Number of days during the 20 day precision trial for which no exclusions were made as a result of obvious faults**

| Analysis | Low Serum | | Medium Serum | | High Serum | |
|---|---|---|---|---|---|---|
| | SP 120 | SMA 12/60 | SP 120 | SMA 12/60 | SP 120 | SMA 12/60 |
| Total protein | 20 | 20 | 20 | 20 | 20 | 20 |
| Albumin | 20 | 20 | 20 | 20 | 20 | 20 |
| Calcium | 19 | 20 | 19 | 20 | 19 | 20 |
| Phosphate | 18 | 20 | 19 | 20 | 19 | 20 |
| Cholesterol | 20 | 20 | 20 | 20 | 20 | 20 |
| Iron | 16 | 20 | 16 | 20 | 16 | 20 |
| Urate | 20 | 20 | 20 | 20 | 20 | 20 |
| Creatinine | 20 | 20 | 20 | 20 | 20 | 20 |
| Total bilirubin | 16 | 20 | 16 | 20 | 16 | 20 |
| Alkaline phosphatase | 20 | 20 | 20 | 20 | 20 | 20 |
| LDH | 16 | 20 | 17 | 20 | 16 | 20 |
| AST | 18 | 18 | 20 | 20 | 20 | 20 |

The system shows good *linearity* over the ranges tested for the individual channels. Linearity could be assumed for the SMA 12/60 but was checked in the case of albumin where at first sight the SP 120 results seemed less obviously linear: there was no difference between the two systems. Table 1 gives the slopes and intercepts of the regression lines for each channel together with correlation coefficients R and SD of the points about the fitted line.

Analytical precision was calculated on the results of the 20-day trial of high medium and low sera. Those runs with data due to malfunctioning analytical channels and faulty printouts which were immediately obvious were excluded. This left the data of runs showing their analytical precision and still containing any faults that could go undetected in the course of routine operation. Table 2 gives the number of days for each machine and each level of sera on which no exclusions were made. Table 3 gives the results of intra-batch precision as an average within-day precision value over the 20 day period. Table 4 places the inter-batch precision figures in their context by comparing them with the recommended values for the Technicon post-maintenance performance check.

There is no difference in performance of the urate and lactate dehydrogenase (LDH) channels, and in three others, total protein, calcium and low-level cholesterol (there is no difference at medium and high levels) precision is lower but not to a clinically noticeable degree. Two channels, bilirubin and aspartate transaminase (AST) give results that are noticeably worse at low concentration, and this being limited to the normal range, where the clinical context tolerates wide variation will be balanced against the definite fall-off in analytical precision. Both channels right themselves at the two higher levels. The iron estimation appears precise only in mid-scale, and both ends of the range show very marked deterioration. We feel that the baseline value, which the SP 120 takes, may be interfered with by the early rise curve of the iron channel thus accounting for the lower SP 120 values. The SMA 12/60 iron method (using ferrozine) is a demanding one requiring exceptional attention to its upkeep, and it seems that its present form will not stand up to operation with half the sample at twice the rate.

Four channels show improvement in precision when operated under SP 120 conditions. These are creatinine and inorganic phosophorus, the clinically critical low level of albumin (at higher concentrations the two systems are equivalent) and alkaline phosphatase, particularly at the lower end of our range.

An early impression — or perhaps suspicion — that mid-point precisions might be best, with a general falling-off at the end of ranges, was not confirmed by our results.

This impression does, however, remain in the *inter-batch precision* (Table 5). Here the variations in bilirubin and, again, iron stand out, together with the high-level alkaline phosphatase results. In the day-to-day measurement of AST the SP 120 system is preferable, though it now appears best in the clinically unimportant low range. For the rest, the two systems perform about equally. The levels of performance are generally acceptable except for high-level creatinine and low-level bilirubin, in both of which an inadequate performance in the

**Table 3 Root-Mean-Square value of each day's intra-batch standard deviation (RMS.SD): means and coefficients of variation (as%) for three concentration levels**

| | | | Low | | | Medium | | | High | | |
|---|---|---|---|---|---|---|---|---|---|---|---|
| Analysis | Units | Machine | RMS.SD | Mean | CV | RMS.SD | Mean | CV | RMS.SD | Mean | CV |
| Total protein | g/l | 12/60 | 0.68 | 45.0 | 1.51 | 1.04 | 62.0 | 1.68 | 1.26 | 82.0 | 1.54 |
| Total protein | g/l | SP120 | 0.81 | 45.0 | 1.81 | 1.40 | 62.0 | 2.26 | 1.64 | 82.0 | 1.99 |
| Albumin | g/l | 12/60 | 0.53 | 27.0 | 1.96 | 0.62 | 35.0 | 1.77 | 0.73 | 43.0 | 1.70 |
| Albumin | g/l | SP120 | 0.48 | 27.0 | 1.77 | 0.60 | 35.0 | 1.72 | 0.75 | 43.0 | 1.75 |
| Calcium | mmol/l | 12/60 | 0.029 | 2.02 | 1.45 | 0.032 | 2.43 | 1.32 | 0.035 | 2.82 | 1.25 |
| Calcium | mmol/l | SP120 | 0.043 | 2.05 | 2.08 | 0.057 | 2.44 | 2.33 | 0.064 | 2.85 | 2.25 |
| Phosphate | mmol/l | 12/60 | 0.040 | 0.84 | 4.76 | 0.040 | 1.15 | 3.44 | 0.054 | 2.05 | 2.63 |
| Phosphate | mmol/l | SP120 | 0.029 | 0.81 | 3.64 | 0.034 | 1.14 | 2.98 | 0.035 | 2.10 | 1.66 |
| Cholesterol | mmol/l | 12/60 | 0.08 | 2.3 | 3.48 | 0.08 | 3.20 | 2.59 | 0.15 | 4.50 | 3.31 |
| Cholesterol | mmol/l | SP120 | 0.11 | 2.2 | 5.15 | 0.13 | 3.10 | 4.27 | 0.17 | 4.50 | 3.79 |
| Iron | μmol/l | 12/60 | 0.43 | 18.8 | 2.34 | 0.43 | 26.1 | 1.66 | 0.44 | 35.2 | 1.24 |
| Iron | μmol/l | SP120 | 1.91 | 13.10 | 14.5 | 2.07 | 21.7 | 9.55 | 2.27 | 33.1 | 6.86 |
| Urate | mmol/l | 12/60 | 0.004 | 0.21 | 2.11 | 0.005 | 0.40 | 1.28 | 0.005 | 0.43 | 1.06 |
| Urate | mmol/l | SP120 | 0.005 | 0.21 | 2.12 | 0.006 | 0.39 | 1.41 | 0.006 | 0.42 | 1.43 |
| Creatinine | μmol/l | 12/60 | 6.44 | 105.0 | 6.14 | 1.81 | 340.0 | 0.53 | 1.30 | 577.0 | 0.22 |
| Creatinine | μmol/l | SP120 | 4.91 | 97.0 | 5.07 | 1.56 | 331.0 | 0.47 | 1.10 | 579.0 | 0.19 |
| Total bilirubin | μmol/l | 12/60 | 0.72 | 14.50 | 4.95 | 1.73 | 58.38 | 2.98 | 3.35 | 133.5 | 2.50 |
| Total bilirubin | μmol/l | SP120 | 1.70 | 14.75 | 11.50 | 2.34 | 60.5 | 3.88 | 3.61 | 133.5 | 2.69 |
| Alkaline phosphatase | IU/l | 12/60 | 2.44 | 14.2 | 17.18 | 3.85 | 63.9 | 6.02 | 5.76 | 156.2 | 3.68 |
| Alkaline phosphatase | IU/l | SP120 | 1.59 | 21.3 | 7.45 | 2.27 | 85.2 | 2.66 | 5.29 | 191.7 | 2.76 |
| LDH | IU/l | 12/60 | 2.54 | 120.0 | 2.12 | 2.65 | 227.0 | 1.17 | 2.14 | 358.0 | 0.60 |
| LDH | IU/l | SP120 | 3.63 | 112.0 | 3.24 | 2.58 | 226.0 | 1.14 | 2.09 | 339.0 | 0.58 |
| AST | IU/l | 12/60 | 1.43 | 23.3 | 6.13 | 2.63 | 47.2 | 5.57 | 3.54 | 129.5 | 2.73 |
| AST | IU/l | SP120 | 2.29 | 19.5 | 11.74 | 2.15 | 42.4 | 5.05 | 3.37 | 123.1 | 2.72 |

**Table 4 Inter-batch precision of the two systems based on mean of daily means.** Three sera (Technicon Ltd) at three concentration levels. Their inscribed values IV and values determined by the two systems are given in SI units, together with their variations (as SD). The SD or CV given with each analysis is the maximum acceptable variation for the post maintenance performance checks of the SMA 12/60 agreed by DHSS and Technicon Co Ltd.

| | | Low | | | Medium | | | High | | |
|---|---|---|---|---|---|---|---|---|---|---|
| Analysis | Units | IV | SMA 12/60 | SP120 | IV | SMA 12/60 | SP120 | IV | SMA 12/60 | SP120 |
| Total protein | g/l | 45 | 45 | 45 | 60 | 62 | 62 | 85 | 82 | 82 |
| SD 1.5 | | | 0.6 | 0.8 | | 0.8 | 1.2 | | 1.5 | 1.6 |
| Albumin | g/l | 35 | 27 | 27 | 45 | 35 | 35 | 60 | 43 | 43 |
| SD 1.3 | | | 1.2 | 0.6 | | 0.7 | 0.7 | | 0.9 | 0.8 |
| Calcium | mmol/l | 2.00 | 2.02 | 2.05 | 2.50 | 2.43 | 2.44 | 3.00 | 2.82 | 2.85 |
| SD 0.038 | | | 0.028 | 0.028 | | 0.032 | 0.038 | | 0.033 | 0.055 |
| Phosphate | mmol/l | 0.50 | 0.84 | 0.81 | 1.30 | 1.15 | 1.14 | 2.10 | 2.05 | 2.10 |
| SD 0.33 | | | 0.02 | 0.02 | | 0.03 | 0.03 | | 0.03 | 0.05 |
| Cholesterol | mmol/l | 2.6 | 2.3 | 2.2 | 3.9 | 3.2 | 3.1 | 6.5 | 4.5 | 4.5 |
| SD 0.19 | | | 0.05 | 0.10 | | 0.08 | 0.10 | | 0.11 | 0.12 |
| Iron | μmol/l | | 19 | 12 | | 26 | 21 | | 35 | 33 |
| | | | 0.5 | 2.3 | | 0.6 | 1.9 | | 0.7 | 2.1 |
| Urate | mmol/l | 0.19 | 0.21 | 0.21 | 0.37 | 0.40 | 0.39 | 0.43 | 0.43 | 0.42 |
| SD 0.012 | | | 0.010 | 0.016 | | 0.010 | 0.014 | | 0.007 | 0.008 |
| Creatinine | μmol/l | 90 | 105 | 97 | 340 | 340 | 331 | 620 | 577 | 579 |
| CV 3% at 180 | | | 5.8 | 6.7 | | 1.7 | 1.7 | | 1.3 | 1.6 |
| Total bilirubin | μmol/l | 17 | 15 | 13 | 68 | 59 | 56 | 140 | 127 | 131 |
| SD 1.7 | | | 0.7 | 1.5 | | 1.5 | 2.5 | | 2.9 | 6.0 |
| Alkaline phosphate | IU/l | 25 | 14 | 21.3 | 100 | 64 | 85 | 200 | 156 | 192 |
| SD 5.0 at 200 | | | 1.6 | 2.3 | | 5.0 | 6.1 | | 9.1 | 18.5 |
| LDH | IU/l | 120 | 120 | 112 | 220 | 227 | 226 | 400 | 358 | 359 |
| CV 5% | | | 2.4 | 3.4 | | 1.6 | 1.5 | | 1.4 | 1.8 |
| AST | IU/l | 25 | 24 | 19 | 45 | 48 | 43 | 135 | 130 | 124 |
| SD 2.5 at 100 | | | 3.9 | 1.3 | | 2.9 | 1.5 | | 3.5 | 3.8 |

**Table 5 Ratios of between-day SD values: SD SP120/SD SMA 12/60**

| Analysis | Low concentration SD Ratio | Medium concentration SD Ratio | High concentration SD Ratio |
|---|---|---|---|
| Total protein | 1.33 | 1.50 | 1.07 |
| Albumin | 0.50 | 1.00 | 0.89 |
| Calcium | 1.00 | 1.18 | 1.67 |
| Phosphate | 1.00 | 1.00 | 1.67 |
| Cholesterol | 2.00 | 1.25 | 1.09 |
| Iron | 4.60 | 3.17 | 3.00 |
| Urate | 1.60 | 1.40 | 1.14 |
| Creatinine | 1.07 | 1.00 | 1.22 |
| Total bilirubin | 2.14 | 1.66 | 2.07 |
| Alkaline phosphatase | 1.44 | 1.22 | 2.03 |
| LDH | 1.31 | 0.92 | 1.29 |
| AST | 0.33 | 0.52 | 1.09 |

SD ratios in the range 0.70 to 1.40 show no real difference between the machine for between-day precision. Ratios less than 0.50 give strong indication that the SP 120 has less day-to-day variation, and ratios above 2.00 indicate that the SP 120 has greater day-to-day variation than the 12/60.

**Table 6 Carryover. Mean I% values of high-low and low-high sequences/and the number of negative I% results in each group**

| Analysis | SMA 12/60 High-low | SMA 12/60 Low-high | SP120 High-low | SP120 Low-high |
|---|---|---|---|---|
| Total protein | 3.14/1 | 3.61/0 | 3.72/1 | 2.95/3 |
| Albumin | 2.89/1 | 4.09/1 | 2.04/0 | 3.29/4 |
| Calcium | 1.38/1 | 1.31/3 | 2.65/0 | 3.71/5 |
| Phosphate | 1.70/1 | 2.29/1 | 1.45/5 | 1.41/5 |
| Cholesterol | 4.47/0 | 4.45/1 | 6.33/0 | 5.95/2 |
| Iron | 2.64/0 | 0.80/0 | 3.00/0 | 3.03/0 |
| Urate | 1.08/0 | 1.97/0 | 1.78/1 | 2.89/0 |
| Creatinine | 1.31/1 | 1.19/1 | 1.72/0 | 2.26/1 |
| Total bilirubin | 1.67/0 | 3.42/4 | 1.03/7 | 4.51/7 |
| Alkaline phosphatase | 3.29/0 | 3.13/0 | 1.62/1 | 1.66/3 |
| LDH | 1.46/0 | 3.83/6 | 1.95/2 | 2.86/6 |
| AST | 2.90/0 | 2.52/0 | 3.84/3 | 4.17/4 |

SMA 12/60 method is further sharpened under SP 120 conditions. The same sharpening effect is seen in the iron method.

Neither intra-batch nor day-to-day precision data give any indication of a significant *drift* effect in routine running conditions but they do not disprove its presence. The SP 120 system improves the intra-batch analysis of AST, an SMA 12/60 channel susceptible to drift; given the same number of samples, the improvement may be due to the shortened SP 120 running time halving the time available for drift to develop.

Our observations on *carryover* are recorded in Table 6. There were 14 high - low or low - high sequences for each channel on each system, with a few instances (bad traces on the SMA 12/60 or error messages on the SP 120) excluded. Carryover is expressed in terms of Young and Gochman's [8] I% interaction value. Broughton et al [1] give 2% as the usual value for I% and regard levels over 5% as unacceptable. None of our SMA 12/60 means reach this figure, and only one SP 120 does, that for cholesterol. This method already has the highest carryover in the SMA 12/60 system, and SP 120 conditions bring out this effect in both the high - low and low - high sequence. Although cholesterol is known as a difficult method, speeding up the analysis has apparently affected only the inter-reaction between viscous solutions, not its precision.

Negative values in the SMA 12/60 system probably have random causes; additional instances amongst the SP 120 results suggest over-correction by the inter-reaction calculation, and there are sixty cases in our SP 120 compared to twenty-three in the SMA 12/60 series. Even in its present form, however, the formula keeps carryover to an acceptable level inside the 5% limit.

### Discussion

Our teething troubles have been limited to the programming (and in particular its response to mechanical events in the SMA 12/60). Low concentrations on two channels (bilirubin and LDH) were on two occasions printed out as zero. On one occasion failure of a colorimeter lamp spoilt two channels, but no error message was printed. An air bubble passing through the flow cell at a critical moment during Initial Tray readings may give a wrong phasing time, faulty inter-reaction calculation, or a high blank leading to falsely low results. Such a mistake occurred three times without an error message showing it, once while the bubble passing through the flow cell was observed on the oscilloscope. Conversely, on one occasion error messages were printed, though the oscilloscope had indicated normal function. A highly efficient debubbler or a further refinement in software is needed to exclude this possibility.

The instrument can print a wider range of results, at least twice that of the SMA 12/60 chart on some channels. The four optical density ranges expand the scale to an extent where the need to reanalyse above-scale samples almost disappears. Apart from a longer setting-up time and some deterioration in results (though improvement in others) the complex generally scores and justifies the changes in operation. It can deal with an increased turnover in that its optimal daily load is higher and for medium-sized hospitals half-day running becomes practicable. It reduces errors in patient identification and its halving of test sample volumes becomes a financial saving on standard and control sera.

An SP 120 run is less labour intensive than its SMA equivalent since no resetting of drift standard is required and the printed patient identities do away with worksheet preparation. The daily manual phasing procedure is also taken over entirely by the SP 120 program.

The complex is well adapted to operate routine SMA methods, but where they operate close to critical variables, these are magnified under SP 120 conditions. In particular, the Liebermann - Burchard-based cholesterol method requires added care, and the ferrozine iron method should not be installed. With these limitations, the SP 120 complex is a successful adaptation of the widely used SMA series of analysers.

The evaluation was carried out between January and June 1977. The manufacturers were informed of the teething troubles listed in this discussion and took these into account in subsequent modifications.

ACKNOWLEDGEMENTS

The authors thank the Department of Health and Social Security for providing the system to be tested, Mr W. I. R. Martin, AWRE, Aldermaston, for the statistical calculations and the staff of Vickers Limited Medical Engineering for their co-operation.

REFERENCES

[1] Broughton, P. M. G, Simpson, D, Mitchell, F. L, Toothill, C, and Whitby, L. G. (1968) *Clinica Chimica Acta* **19**, 297-311

[2] Broughton, P. M. G, Gowenlock, A. H, McCormack, J. J, and Neill, D. W. (1974) *Annals of Clinical Biochemistry*. **11**, 207-218

[3] Schwartz, M. K, Bethune, V. G, Fleisher, M, Pennacchia, G, Menendez-Botet, C. J. and Lehman, D. (1974) *Clinical Chemsitry* **20**, 1062-1070

[4] Technicon Bibliography 1967-1973. Technicon Instruments Corporation, Tarrytown, New York.

[5] Northam, B. E, Widdowson, G. M. Association of Clinical Biochemists, Scientific and Technical Committee, *Technical Bulletin No 11*, Oct. 1967

[6] Robinson, R, Roughan, Mary E, Wagstaff, D. F. (1971) *Annals of Clinical Biochemistry* **8**, 168-170

[7] Goldberg, D. M, Benton, J. M, Scott, Jennifer, Stelling, D. *Technicon England, Symposium* 1971, 83-94

[8] Young, D. S. Gochman, N. (1972) *Standard Methods in Clinical Chemistry* **7**, 293-304

